# Spatio-temporal variation in number and production of neustonic and planktonic bacteria inhabiting polluted estuarine harbour channel

**DOI:** 10.1007/s00203-021-02538-6

**Published:** 2021-08-25

**Authors:** Piotr Perliński, Zbigniew J. Mudryk, Marta Zdanowicz, Łukasz Kubera

**Affiliations:** 1 Department of Experimental Biology, Institute of Biology and Earth Sciences, Pomeranian University in Słupsk, Arciszewskiego 22b, 76-200, Słupsk, Poland; 2 Department of Microbiology and Immunobiology, Faculty of Biological Sciences, Kazimierz Wielki University, Al. Powstańców Wielkopolskich 10, 85-090 Bydgoszcz, Poland

**Keywords:** Harbour channel, Surface microlayer, Subsurface water, Total bacterial number, Bacterial production

## Abstract

The aim of this paper was to determine the abundance and secondary production by bacteria inhabiting the surface microlayer and subsurface water in a specific water basin, i.e., polluted estuarine harbour channel. In a 3-year seasonal cycle, the total number of bacteria and their biomass were higher in the surface microlayer (SML) 7.57 × 10^8^cells dm^−3^ and 15.86 µg C dm^−3^ than in the subsurface water (SSW) 4.25 × 10^8^cells dm^−3^ and 9.11 µg C dm^−3^ of the studied channel. The opposite relationship was noted in the level of the secondary production (SML—37.16 μg C dm^−3^ h^−1^, SSW—60.26 μg C dm^−3^ h^−1^) in this water basin. According to the analysed microbiological parameters, the total number of bacteria and secondary production varied along the horizontal profile in the water of the studied channel. The total number of bacteria and their secondary production showed the seasonal variation as well.

## Introduction

The surface microlayer (SML) is a boundary between the hydrosphere and atmosphere of a thickness varying from 10 to 250 µm, which chemical, physical, and biological properties differ from those of the subsurface water (SSW) (Chance et al. 2018; Zäncker et al. 2021). The SML is one of the largest and most important interface on our planet and it occupies approximately 70% of the Earth’s surface, covering all marine, estuarine, and freshwater bodies (Astrahan et al. 2016; Engel et al. 2017). The SML is a gel-like proteinaceous hydrated elastic biofilm that is composed mainly of transparent exopolymer particles (Engel and Galgani 2016; Dreshchinskii and Engel 2017). This boundary layer constitutes one of the most geochemically and biologically active areas of the biosphere (Ram et al. 2018). The SML is biochemical microreactor, which plays a key and unique role in the dynamic exchange of various gases, mainly CO_2_, and heat between the hydrosphere and atmosphere; it has potentially significant effects on climate change, regulation of biogeochemical cycles on global scale, precipitation, temperature changes, and controls the rates of exchange of energy and matter between water basins and air (Sabbaghzadeh et al. 2017; Wurl et al. 2017). Although the SML as a very dynamic structure is frequently disrupted by rain and wind of speed greater than 5–6 m s^−1^ (Engel and Galgani 2016; Sun et al. 2018), the results of stirred tank experiments suggest that microlayers can self-reconstruct their original structure in less than a minute (Cunliffe et al. 2013). The surface microlayer is generally enriched in both dissolved and particulate organic matter by physical processes including diffusion, convection, advection, adhesion to rising bubble, upwelling of sub-surface water and particle deposition from air (Kurata et al. 2016; Perliński et al. 2017). Compared to subsurface waters, the surface microlayer can be enriched up to10^2^–10^3^ times in molecular and dissolved organic matter consisting mainly of proteins, carbohydrates, lipids, nucleic acids, chitin, pectin and cellulose (Perliński et al. 2017). Such biogenic molecules have been shown to be major biochemical components of the SML (Galachyants et al. 2018; Shaharom et al. 2018). The primary sources of these organic compounds are phytoplankton and bacteria exudates, terrestrial organic matter transported from land, natural organic matter from the water column and bottom and various anthropogenic sources (Engel et al. 2017; Sabbaghzadeh et al. 2017). The enrichment of biogenic organic matter can render the SML to favourable habitat to development of microbial life (Zäncker et al. 2017). It is worth noting that apart from the factors positively influencing the number of microorganisms in the surface microlayer, we can also observe the influence of negative factors, such as temperature amplitude, solar radiation (particularly UV) and chemical toxic substances, as well as mortality by viruses and grazing (Antonowicz et al. 2017a). For example, a study conducted by Tovar-Sánchez et al. (2014) revealed that the SML of the Mediterranean Sea is enriched with bioactive trace metals ranging from 8 (for Cd) to 1000 (for Fe) times higher than the dissolved metal pool in the subsurface water.

Life in this unique ecotone is dominated by different microorganisms called neuston (Kostrzewska-Szlakowska and Kiersztyn 2017; Helm 2021). An extensive research has shown that the surface microlayer contains elevated number of heterotrophic and autotrophic microorganisms including mainly bacteria, cyanobacteria, flagellates, algae, and invertebrates (Santos et al. 2011; Astrahan et al. 2016). Among these neustonic organisms, bacterioneuston is dominant, for which the SML is considered favourable life habitat due to the accumulation of a large amount of organic matter (Kostrzewska-Szlakowska and Kiersztyn 2017; Antonowicz and Kozak 2020). This bacterial community plays a considerable role in maintaining the SML physicochemical properties, is actively involved in the exchange of substances and gases, mainly greenhouses gases, between the atmosphere and hydrosphere and is essential to the global organic matter cycle (Cunliffe et al. 2011; Kurata et al. 2016; Galachyants et al. 2018). According to Zwisler et al. (2003) and Franklin et al. (2005) to fully understand the role of bacterioneuston in the function of the surface microlayer, it is necessary not only to determine its enzymatic activity, physiological properties and taxonomic diversity of these organisms but also determine its abundance and level of the secondary production. Hence, during recent years, numerous studies on the number and productivity of bacterioneuston in freshwater (Kalwasińska and Donderski 2005; Zdanowicz and Mudryk 2017; Galachyants et al. 2018), estuarine (Mudryk and Skórczewski 2007; Santos et al. 2011; Azevedo et al. 2012), and marine environments (Stolle et al. 2010; Nakajima et al. 2013; Astrahan et al. 2016) were carried out. However, to our knowledge, no studies on the abundance of neustonic bacteria and their secondary production rate in a specific water reservoir such as an estuarine harbour channel are available so far. Therefore, the aim of this paper was to determine spatio-temporal heterogeneity in the abundance and level of carbon production by bacteria inhabiting the surface microlayer and subsurface water in the estuarine channel in the port Ustka (southern Baltic Sea).

## Materials and methods

### Study area and sampling

**Fig. 1:**
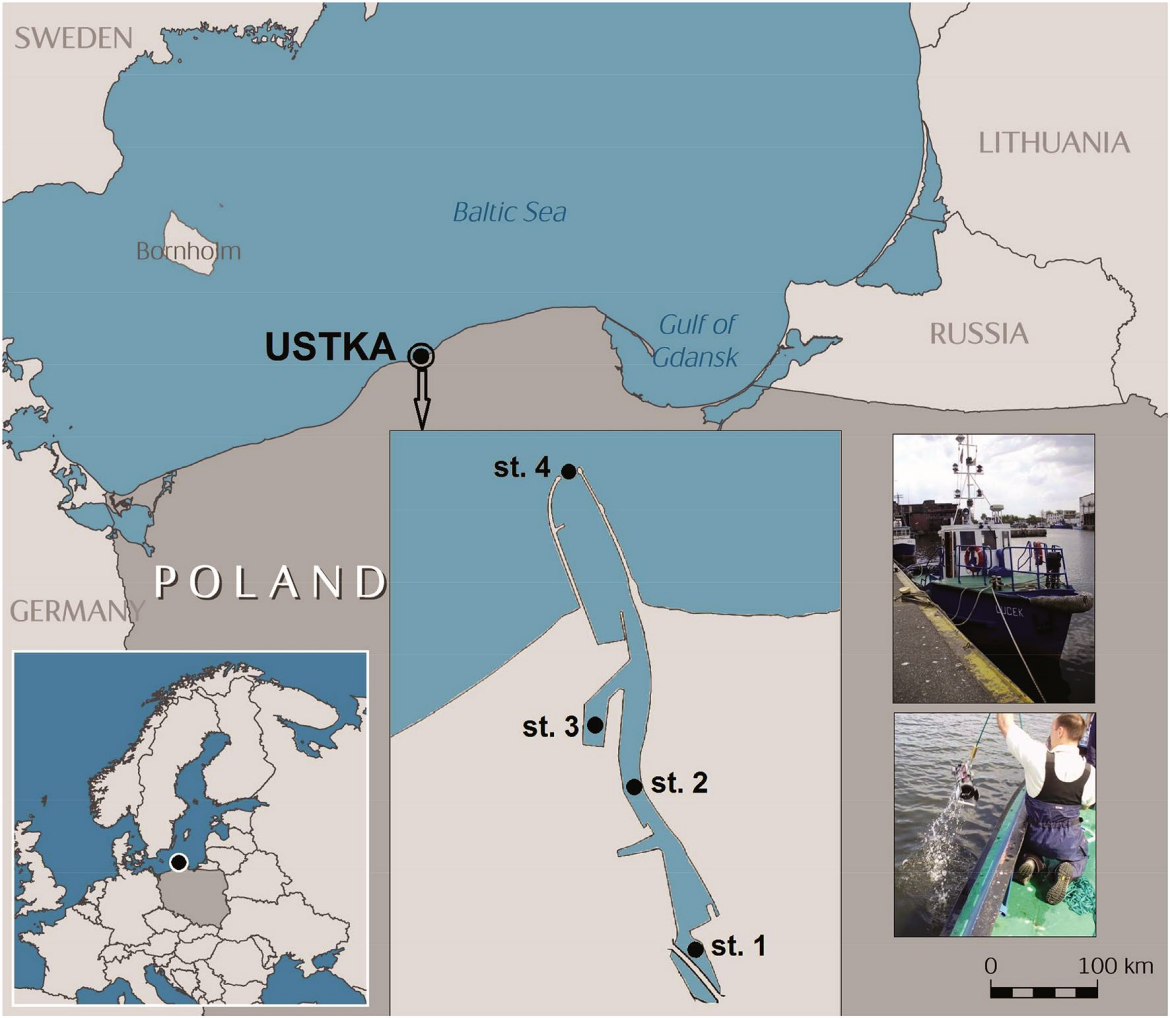
Map of localization the study estuarine harbour channel and sampling sites in Ustka

This study was carried out in the harbour channel (Fig. 1), which is the estuarine part of the Słupia River (Poland). The catchment area of the river covers 1623 km^2^ over 60% of that area is exploited mainly for agricultural purposes (Perliński et al. 2017). This river carries 15.5 m^3^ s^−1^ of water into the Baltic Sea, as well as 200,000–300,000 m^3^ year^−1^ of natural and anthropogenic sediments (Zawadzka 1996). The studied channel is 1.1 km long, 40.5 m wide and about 6 m deep, and is located in the vicinity of the port in Ustka (54° 35.2 N, 16° 21.2 E) (Fig. 1). The studied harbour channel is limited by two breakwaters of about 300 m length, which are also the final part, where the Słupia River enters the sea (Perliński et al. 2017). The port in Ustka covers the area of 0.3 km^2^ and its main functions are fishery, marine transport, tourism, and shipbuilding (Christowa et al. 2007). All these economic activities bring a significant load of contamination, in particular, heavy metals, which concentration in the water of the port and coastal sea water in its region is presented in Table 1. At the same time, this harbour channel is the home port for 113 vessels. All these vessels (such as fishing boats, yachts and passenger ships) are the sources of petroleum hydrocarbon pollution, which concentration in the years 2005–2017 varied in the range of < 0.1 to 1.45 g m^−3^ according to the Maritime Office in Słupsk.

**Table 1:** Mean concentrations of heavy metals and arsenic and standard deviations recorded in surface waters of the Harbour and coastal sea waters (Antonowicz et al. 2017b)

Chemical parameters	Harbour		Coastal sea waters	
	Mean	SD	Mean	SD
	Concentration (μg dm^−3^)			
Mn	2.66	1.09	3.07	2.16
Ni	4.50	4.08	10.93	2.87
Cu	3.56	2.89	5.5	2.43
Zn	< 20	–	< 20	–
Cd	0.14	0.81	0.21	0.08
Pb	< 5	–	< 5	–
As	0.41	0.38	0.62	0.71

Selected chemical and microbiological parameters of the water in the harbour channel are presented in Table 2.

**Table 2:** Values of selected chemical and microbiological parameters in the harbour channel (Perliński 2015)

Parameters	Units	st.1		st.2		st.3		st.4	
		Mean	Range	Mean	Range	Mean	Range	Mean	Range
N–NO_3_	mg dm^−3^	7.26	1.37–15.98	8.49	3.65–15.66	13.56	3.9–48.43	11.68	4.16–22.3
N–NH_4_	mg dm^−3^	48.45	26.06–103.23	50.39	25.27–136.43	43.82	22.4–100.25	45.93	21.97 –106.63
Cl^−^	mg dm^−3^	294.28	36.46–741.40	419.57	94.58–1250.28	574.87	128.88–1306.12	1852.13	381.92–5909.11
O_2_	mg dm^−3^	7.05	4.76–10.42	7.44	4.17–11.11	6.81	5.63–7.61	7.06	5.21–9.13
pH		7.28	6.80–7.70	7.27	6.53–7.70	7.45	7.09–7.70	7.45	7.18–7.67
OM	mg dm^−3^	0.45	0.30–0.70	0.79	0.30–1.70	0.68	0.40–0.90	0.89	0.20–2.30
Protein	µg dm^−3^	23.81	15.12–34.32	22.10	16.31–28.01	21.01	15.31–27.42	22.99	17.31–29.87
Lipids	µg dm^−3^	109.21	43.88–222.57	85.21	36.27–175.54	105.04	24.38–248.46	360.57	20.09–888.59
Carbohydrates	µg dm^−3^	72.31	31.27–135.11	75.46	21.87–154.96	60.74	32.03–91.13	47.33	24.36–70.55
Number of heterotrophic bacteria	10^6^ dm^−3^	23.30	3.17–91.67	23.40	2.33–79.18	24.50	4.00–79.18	19.17	1.50–62.27
Heterotrophic bacterial biomass	mg dm^−3^	37.41	5.07–146.67	37.44	3.73–126.67	39.20	6.40–126.67	30.68	2.40–100.27

The water samples from the channel in Ustka were collected at four sites (Fig. 1).

Site 1—located on the border between the Słupia River and the studied channel.

Site 2—placed in the central part of the channel.

Site 3—located in one of the water basins called the coal basin.

Site 4—located at the site, where the estuarine channel enters the sea, i.e., near the heads of breakwaters.

The precise location of each sampling site was taken with a GPS receiver installed on the board of the tugboat (Fig. 1). The water samples were collected quarterly in autumn (a), winter (w), spring (sp), and summer (s) during 2010–2013. The surface microlayer (SML) samples (thickness 150–250 μm) were collected with a 75 × 75 cm metal screen according to the method of Garrett (Garrett 1965). The mesh size of the metal screen was 0.14 mm. The water collected with the Garrett net was scrapped off with the wiper and then transferred to a sterile bottle. The subsurface water (SSW) was collected at about 0.5 m depth with a horizontal van Dorn water sampler adapted for the collection of the samples in flowing water (Fig. 1) (Perliński et al. 2017). The collected water samples were transferred to sterile bottles using drain valve. Prior to sampling, the Garret net and van Dorn sampler were rinsed several times with distilled sterile water and 80% ethyl alcohol. The collected samples of water were transported to the laboratory in the ice containers at the temperature that did not exceed 7 °C.

### Determination total bacteria number and biomass

The total bacterial number (TBN) was established with the DAPI (4,6-diamidino-2-phenyl-indol) direct method according to Porter and Feig (1980). Aliquots of 10 cm^3^ were preserved with formaldehyde at final concentration of 1%. A solution of DAPI ≥ 98% (Fluka) at a concentration of 50 μg cm^3^ was added to 1 cm^3^ of the fixed water sample. After mixing the sample on a shaker, it was placed in the dark for 10–15 min to stain bacterial cells. Next using a Millipore filtration apparatus stained samples were filtrated with black—stained polycarbonate filters (Millipore) (0.2 μm pore size, 13 mm diameter). After filtering the sample, the filters were rinsed with 1 cm^3^ of 80% ethyl alcohol and then with 2 cm^3^ of double-distilled water. Counting bacteria absorbed on the filters were performed at 1000 × magnification using OLYMPUS BX–41 epifluorescence microscope (equipped with an excitatory—barrier block UV-2A—excitation *λ* = 365 nm, emission *λ* = 420 nm) coupled with a Colour View III camera. Bacteria stained in blue were counted in 20 different fields with a minimum of 200 cells. TBN was calculated according to the formula proposed by Zimmermann and Meyer-Reil (1974). Bacterial cell numbers were transformed to bacterial biomass (BB) using the conversion factor 20 fg C cell^−1^ (Lee and Fuhrman 1987; Chróst et al. 2000).

### Estimated bacterial production

The secondary production of bacteria (BP) in the water samples was determined by measuring the rate of incorporation of methyl—[^3^H] thymidine ([^3^H]TdR) into the bacterial DNA (Fuhrman and Azam 1982; Mudryk and Skórczewski 2007). To determine this parameter, 20 μl [^3^H]TdR (NEN Life Science Products 60 Ci nmol^−1^ specific activity) was added to 10 cm^3^ water samples in three replications with final concentration of 20 nM dm^−3^ and the samples were incubated for 60 min at 20 °C. After this time, the incubation was stopped by adding 200 μl of 37% formaldehyde to the samples. A prekilled sample was used as a blank. The samples were then filtered with a Millipore sampling manifold on 0.22 μm nitrate cellulose filters (Millipore, 25 mm diameter). The filters were rinsed twice with 5 cm^3^ 10% ice-cold TCA and then dissolved in 1 cm^3^ of ethyl acetate and placed in scintillation vials (Packard) with 10 cm^3^ LCS-cocktail (Packard, Filter-Count). After 24 h, the samples were radio-assayed in a Packard TRI—CRAB 2100TR liquid scintillation counter. The calculation of bacterial production was based on thymidine incorporation (TdR) using a factor of 1.25 × 10^9^ cells nmol^−1^ thymidine (Chróst et al. 1988). The bacterial growth rates (μ) were estimated by dividing bacterial production (µg C dm^−3^ h^−1^) with biomass (µg C dm^−3^) following Grossman and Dieckmann (1994), and Nakajima et al. (2013).

### Statistical analysis

Standard statistics of data variation and dispersion (standard deviation—SD, coefficient of variation—CV, coefficient of dispersion—CD) were calculated based on Velji and Albright (1986).

Enrichment or depletion of the studied bacterial parameters in the SML and SSW was estimated by calculating the average enrichment factor (EF). EF = *x* (SML)/*x* (SSW), where “*x*” was the value of a given bacteriological parameter in the SML or SSW, respectively. EF value > 1.0 is defined as enrichment, while EF value < 1.0 as depletion (Engel et al. 2017).

Following Incera et al. (2003), the significance of difference between the studied bacterial parameters among the layers, sites and seasons was assessed by a two-way ANOVA of variance and the Kruskal–Wallis test, non-parametric equivalent of ANOVA, when the data distribution differed from a normal distribution.

Simple linear regressions were carried out to determine the relationship between the total bacterial number and bacterial production in the surface microlayer and subsurface water (Zdanowicz and Mudryk 2017).

## Results

The total number of bacteria (TBN), their biomass (BB), bacterial secondary production (BP), and bacterial growth rate (*µ*) in a 3-year seasonal cycle in the water of the studied channel are presented in Table 3. The analysis of these data showed that the total number of bacteria in the studied water layers varied in different years from 0.18 to 14.72 cells × 10^8^ dm^−3^ with the mean value of 5.91 cells × 10^8^ dm^−3^, while their biomass changed from 2.86 to 29.44 μg C dm^−3^ with the mean value of 12.48 μg C dm^−3^. The bacterial secondary production (BP) estimated from the rate of thymidine incorporation in the bacterial DNA in the studied channel varied from 0.86 to 222.00 μg C dm^−3^ h^−1^ (mean 48.71 μg C dm^−3^ h^−1^) and the bacterial growth rate changed within the range 0.04–32.71 day^−1^ (mean 4.9 day^−1^). Data presented in Table 3 show that the total number of bacteria and their biomass enrichment factor were higher (EF_TBN,BB_ = 1.7–1.8) in the surface microlayer (SML) than in the subsurface water (SWW). In contrast, the rate of secondary production of bacteria and bacterial growth rates was higher (EF_BP,µ_ = 0.4–0.6) in the subsurface water compared to the surface microlayer.

**Table 3:** Total number bacteria, biomass bacteria, bacterial production and bacterial growth rates in studied harbour channel (average data from three years of research)

Bacterial parameters	Layer	Mean	Range	SD	CV [%]	CD	EF
Total bacterial number [10^8^ dm^−3^]	SML	7.57	0.43–14.72	3.39	44.75	1.52	1.8
	SSW	4.25	0.18–8.96	2.08	48.86	1.01	
Bacterial biomass [µg C dm^−3^]	SML	15.86	6.80–29.44	3.59	22.6	0.81	1.7
	SSW	9.11	2.86–17.91	4.88	53.5	2.61	
Bacterial production [μg C dm^−3^ h^−1^]	SML	37.16	0.86–205.51	50.83	136.8	69.51	0.6
	SSW	60.26	7.58–222.00	53.59	88.9	47.65	
Bacterial growth rate (*µ*) [day^−1^]	SML	2.55	0.04–13.29	3.56	139.48	4.96	0.4
	SSW	7.25	0.68–32.71	5.30	73.13	3.88	

**Fig. 2:**
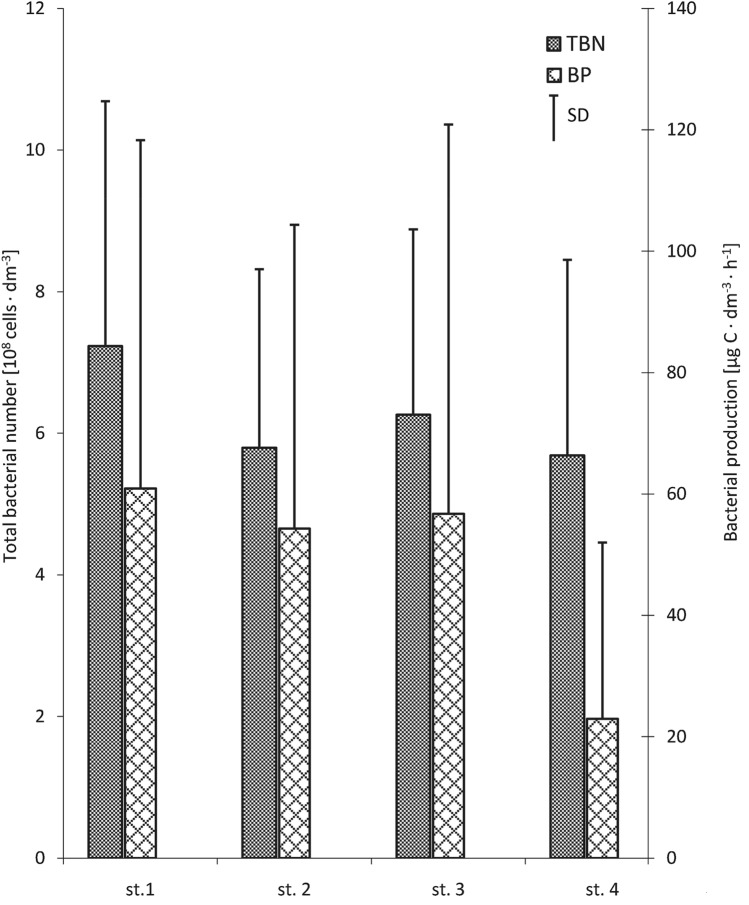
Horizontal variations of total bacterial number and production in studied channel (average from the pooled data of both water layers and all seasons). Vertical bars represent standard errors of the mean

The results of the present study indicated the difference in the total number of bacteria and secondary production along the horizontal profile in the water of the studied channel (Fig. 2). The highest total number of bacteria was noted on the border between the Słupia River and the studied channel (st.1) (7.23 cells × 10^8^ dm^−3^), while in the remaining sites, it was much lower and at a similar level (5.68–6.26 cells × 10^8^ dm^−3^). The bacterial secondary production also varied between the zones of the harbour channel in Ustka. The level (22.95 μg C × dm^−3^) of secondary production of bacteria at the site located where the channel enters the sea (st.4) was two to three times lower than at other sites (54.28–60.89 μg C × dm^−3^).

**Fig. 3:**
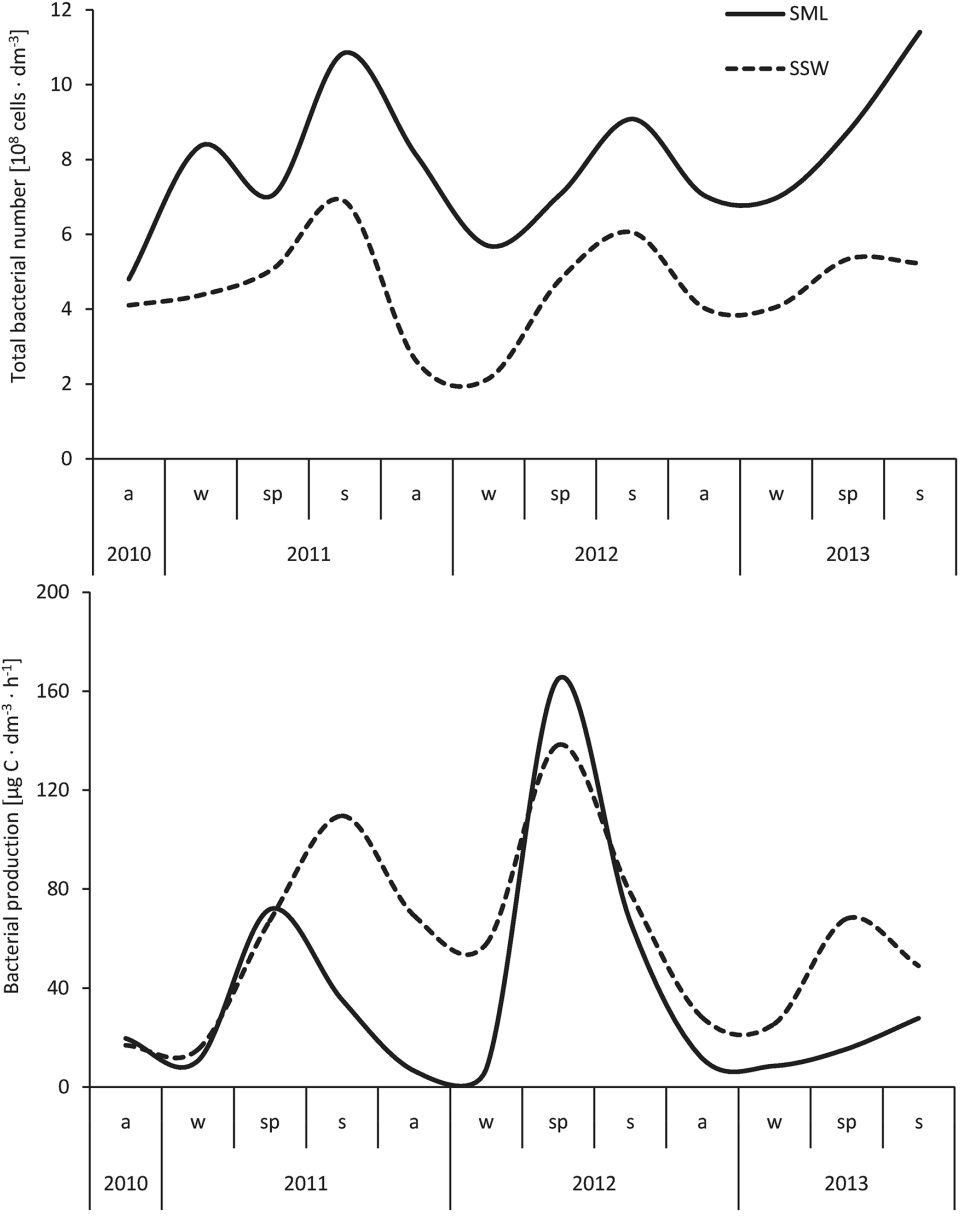
Seasonal dynamics change of total bacteria number and bacterial production in surface microlayer and subsurface water in channel in Ustka (average from the pooled data of all sites)

The total number of bacteria in the surface microlayer and subsurface water layers changed with the seasons. Data presented in Fig. 3 show that in a three-year seasonal cycle, the maximum values of the TBN in the surface microlayer (11.40 cells × 10^8^ dm^−3^) and subsurface layers (6.88 cells × 10^8^ dm^−3^) were recorded in summer. The minimum numbers (4.80 cells × 10^8^ dm^−3^) of bacterioneuston were noted in autumn and of bacterioplankton in winter (2.13 cells × 10^8^ dm^−3^). Large fluctuations of the seasonal dynamics of the level of bacterial production were also documented in the studied water layers (Fig. 3). In the surface microlayer (164.82 μg C dm^−3^ h^−1^) and subsurface water (138.20 μg C dm^−3^ h^−1^), the maximum level of carbon production by bacteria was noted in the spring and summer seasons. The minimum rate of the secondary production of bacterioneuston (7.16 μg C dm^−3^ h^−1^) and bacterioplankton (15.14 μg C dm^−3^ h^−1^) was recorded in winter.

**Fig. 4:**
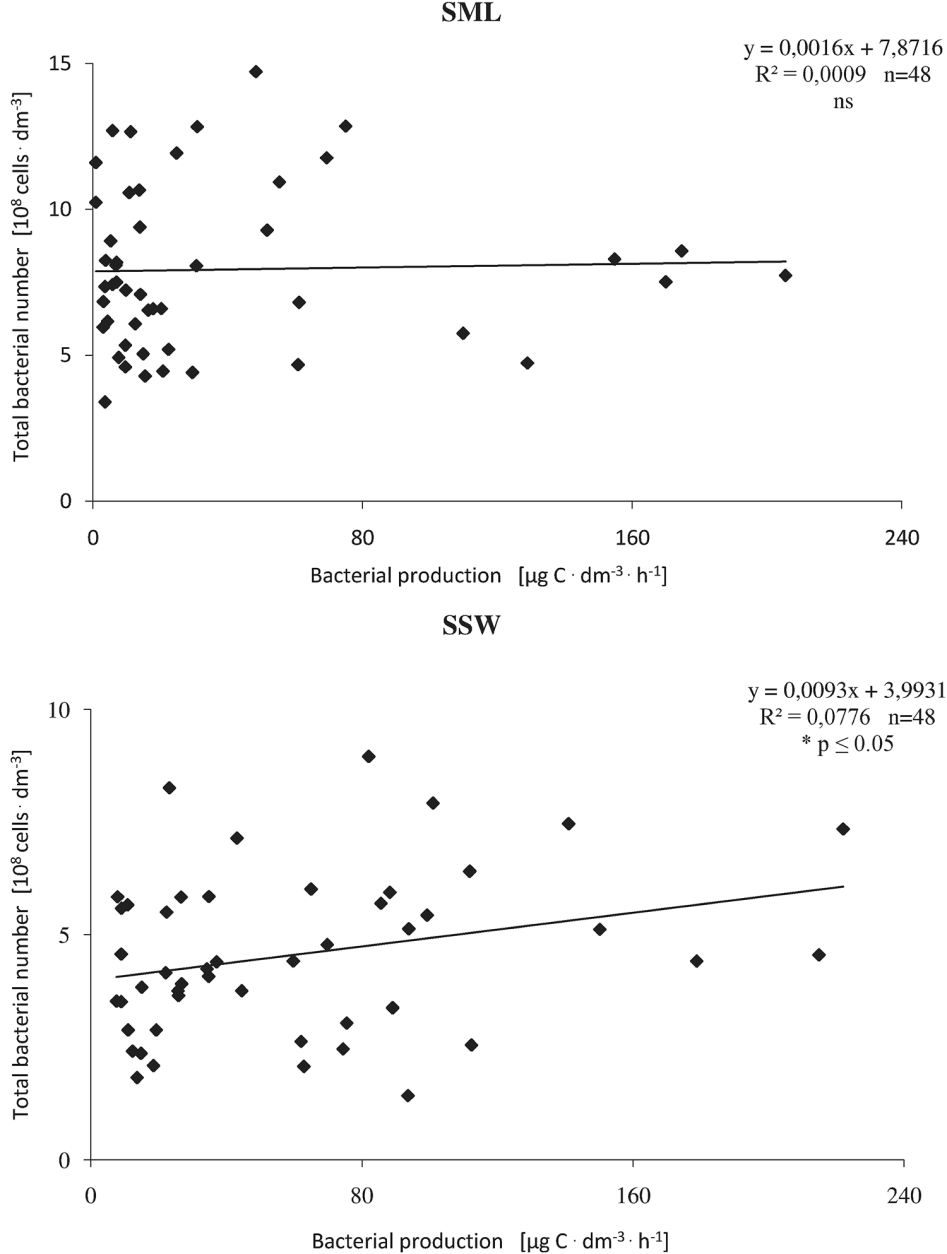
Relationship between total bacteria number (TBN) and level bacterial production (BP) in surface microlayer (SML) and subsurface water (SSW) in harbour channel. Solid lines represent linear regression including all data (*y*- regression equation *R*^2^-coefficient of determination, *n* number samples, *p* significant level)

Linear regression showed that there was no significant relationship between the TBN and TB in the surface water. The highest correlation (*R*^2^ = 0.077, *p* < 0.05) was found between the TBN and BP in the subsurface water (Fig. 4).

The obtained results were grouped by the seasons, layers and sites, and a two-way factorial ANOVA test was carried out for the total bacterial number and bacterial production (Table 4). The analyses showed significant differences in the total bacterial number among the layers and seasons, while there were no significant differences among the sites. The bacterial productivity rates were high (*p* < 0.001) and significantly differ (*p* < 0.05) between the seasons and layers, while the difference between the sites was low.

**Table 4:** Analyses of two-way-ANOVA rang Kruskal–Willis of variance in the studied taxonomic groups**,** due to site, layer and season

Parameters	Source of variation	*H*	*p*
TBN	Site	3.163	ns
	Layer	35.273	***
	Season	17.495	***
	Site × layer	39.559	***
	Site × season	26.060	*
	Layer × season	53.208	***
BP	Site	10.739	*
	Layer	11.861	***
	Season	22.625	***
	Site × layer	29.524	***
	Site × season	37.783	***
	Layer × season	36.949	***

*ns* non significant, *H* the Kruskal–Wallis test, *p* significance level

Significance (*p*) is indicated by asterisks *p < 0.05, ***p < 0.001

## Discussion

Bacteria play a major role in functioning of aquatic ecosystems by actively participating in metabolic conversions of various forms of organic matter, recycling of nutrients and energy flux (Yucel 2017; Figueroa et al. 2021). Hence, the basic parameters used to describe the structure and metabolic activity of bacteriocenosis in all water basins are the total number and productivity of bacteria (Ameryk et al. 2005; Kostrzewska-Szlakowska and Kiersztyn 2017). This parameter is essential in our understanding of their ecological role and their contribution to marine processes.

The total bacterial number in Ria de Averio, a tidal lagoon (western coast of Portugal), varied from 0.2 to 8.5 × 10^9^ cells dm^−3^ (Almeida et al. 2001), while in Kiel Bight (Baltic Sea), it was 2.8 × 10^9^ cells dm^−3^ (Mock et al. 1997). Much lower total bacterial number (0.43–14.72 × 10^8^ cells dm^−3^) was documented in the water of the studied harbour channel in Ustka. This low number of bacteria probably results from pollution of this water basin with heavy metals and petroleum hydrocarbon pollutants. This hypothesis is confirmed by Ma et al. (2015) and Xu et al. (2018).

An important mechanism for the transformation of organic matter accumulated in water bodies is the secondary production of bacteria (Steinberg et al. 2008; Santos et al. 2013). The calculation of this parameter enables estimation of the amount of organic matter transferred between the different links in the trophic chain in aquatic ecosystems (Hyun and Kim 2003; Lemée et al., 2002). In the water of the studied estuarine harbour channel, the secondary production of bacteria varied from 37.16 to 60.26 μg C dm^−3^ h^−1^. This was higher than in the Ria de Averio tidal lagoon (0.05–4.5 μg C dm^−3^ h^−1^) (Almeida et al. 2001), in the Scheldt estuary (0.3–11.5 μg C dm^−3^ h^−1^; Goosen et al. 1995), and in the Baltic Sea (Kiel Bight) (0.021–0.235 µg C dm^−3^ h^−1^; Mock et al. 1997). While, the level of bacterial secondary production in the channel in Ustka was significantly lower than those reported by La Ferla et al. (2005) in the Central Mediterranean Sea.

The results of the present study on the total number of bacteria inhabiting analysed water layers in the estuarine harbour channel in Ustka showed that this parameter was almost two-fold higher in the surface microlayer (7.57 × 10^8^ cells dm^−3^) than in the subsurface water (4.25 × 10^8^ cells dm^−3^). The same pattern was also documented by the studies on bacterioneuston and bacterioplankton abundance in inland reservoirs (Zdanowicz and Mudryk 2017; Galachyants et al. 2018), estuaries (Santos et al. 2009, Azevedo et al. 2012), and marine environment (Nakajima et al. 2013; Fan et al. 2018). Many abiotic and biotic factors influence such numerous occurrence of bacteria in the surface microlayer. The accumulation of significant amounts of organic compounds potentially bioavailable for consumption by live organisms in the surface membrane stimulates optimal conditions for the development of bacterial microflora (Cunliffe et al. 2013; Astrahan et al. 2016). Also, an important driving force generating large amounts of bacterioneuston in the air–water boundary layer are excretions and secretions of phyto- and zooneuston consisting mainly of carbohydrates, proteins, and lipids (Wurl and Obbard 2004). These compounds are very actively assimilated by neuston bacteria as food substrates used to build cellular structures and as energy material necessary for metabolic processes (Santos et al. 2011; Astrahan et al. 2016). Another factor increasing the number of bacteria in the surface microlayer is the presence of specific extracellular structures in neustonic organisms. These are hydrophobic compounds (mucopolysaccharides, glycoproteins, phosphocholine lecithin), which significantly facilitate the adhesion of neuston bacteria to the surface microlayer (Walczak and Donderski 2005; Zdanowicz and Mudryk 2017). At the same time, many planktonic and benthic bacteria with the ability to move may migrate from deeper layers of water and bottom sediments to the surface microlayer by chemotaxis, flotation, adhesion to rising bubbles and buoyant particles (Stolle et al. 2010; Nakajima et al. 2013). Hervas and Casamayor (2008) also point out that the surface microlayer may also be enriched with bacteria by solid and liquid atmospheric precipitation. According to Antonowicz et al. (2015) and Zdanowicz, and Mudryk (2017), a very good oxygenation of this water layer also provides aerobic bacterioneuston with optimal development conditions.

Our research on the level of bacterial secondary production in the surface microlayer and subsurface water in the studied channel showed that the higher level of bacterial carbon production (EF = 0.6) was found in the subsurface water. According to Zdanowicz and Mudryk (2017), these results indicate that planktonic bacteria are characterized by higher metabolic activity than neustonic bacteria due to their short generation time and high rate of cellular carbon production. Also, previous studies (Kalwasinska and Donderski 2005; Obernosterer et al. 2008; Santos et al. 2014; Antonowicz et al. 2015) documented that the production of bacterioplankton was higher than that of bacterioneuston. Stolle et al. (2009) and Zàncker et al. (2017) reported that thymidine incorporation by bacterioneuston in the Baltic Sea and Pacific Ocean was reduced by 5–80% compared to bacterioplankton. The results of the study by Obernosterer et al. (2008) allowed to estimate the fraction of active bacterial cells for roughly 10% and 25% of DAPI-stained cells in the surface microlayer, respectively.

Low bacterial productivity at the surface microlayer indicates that the air–water interface is a stressful environment for bacteria (Stolle et al. 2009; Santos et al. 2014). According to Kalwaśińska and Donderski (2005) and Obernosterer et al. (2008), low bacterial productivity at the SML is probably caused by the stressogenic effect of environmental factors, mainly drastic changes in temperature, pH, salinity conditions and relatively high accumulation of heavy metals, biophenol, polyvinyl chloride, and pesticides. Those strong toxic compounds are frequently found in the SML in the quantity exceeding concentrations, which are safe for proper functioning of organisms inhabiting the surface microlayer, mainly bacteria algae and fungi (Antonowicz et al. 2017a). This thesis was confirmed by the studies of van Westernhagen et al. (1987) and Wurl and Obbard (2004) indicating that the concentrations of heavy metals in the surface microlayer were 100 times and toxic substances 500 times higher than in the subsurface water layers. The decrease in the level of bacterial production in the SML might be also caused naturally when bacterioneuston is exposed to high levels of solar radiation including the UV spectrum (Zàncker et al. 2017). Ultraviolet may cause photo-damage to cells by catalysing the intracellular formation of chemical intermediates, such as reactive oxygen species (ROS) and DNA photoproducts (Santos et al. 2014; Astrahan et al. 2016). Bacterial metabolic activity is especially sensitive to the ultraviolet UV-B (280–320 nm) and UV-A (320–400 nm) range (Obernosterer et al. 1999). Naganuma et al. (1996) and Benner and Biddanda (1998) found decreased bioavailability of organic matter in bacteria exposed to UV radiation. The exposure of dissolved organic matter to solar radiation leads to the inhibition of bacterial growth and formation of substances which decrease the level of bacterial production (Joux et al. 2006; Zàncker et al. 2017).

The horizontal zonation of bacterial abundance is a well-known global phenomenon reported from different water basins (Varela et al. 2003; Anas et al. 2021; Figueroa et al. 2021). The results of the present study carried out along the horizontal transect in the harbour channel in Ustka also indicated that bacterial abundance was highly variable between the sample sites of the studied water basin. The total number of bacteria over the longitudinal transect was the highest in freshwater (site 1), while it was much lower, but at similar level, at all other sites. Probably this variation in the number of bacteria along the horizontal profile of the harbour channel in Ustka may result from changing water salinity. A gradient of decreasing bacterial numbers with increasing salinity was noted in several water basins (Cunliffe et al. 2009; Lefort and Gasol 2013; Jacquemot et al. 2021) and that corresponds well with our results. According to Santos et al. (2011) in the Ria de Aveiro estuary, total bacterial number was lower in hydrodynamic zone (seawater zone), whereas it was higher in more stable zone (freshwater and brackish water zone). The relation between prokaryotic cell number and salinity in water ecosystems is negative because bacteria require more energy for the production of osmolytes and less for reproduction (Perliński et al. 2017). In addition to salinity, an important factor limiting the number of bacteria in the water in the central and ending part of the harbour channel (Table 4) was the accumulation of heavy metals and a relatively large accumulation of petroleum hydrocarbon pollutants.

Similarly to the total bacteria number, the level of secondary production of bacteria varied between the zones of the studied harbour channel in Ustka. The secondary production of bacteria at the site located where the channel enters the sea was two to three times lower than at other sites. Also Varela et al. (2003) in the Ria de Ferrol (Galicia, NW Spain) noted that bacterial carbon production was higher in the inner part of the estuary than in the outer station. Such differences in the level of bacterial production in the horizontal profile may be explained by the results of the studies carried out by Dagg and Breed (2003) and Manini et al. (2004). These researchers point out that in addition to composition, availability and degradability of organic compounds, originating mainly from primary phytoplankton production, in the river estuaries such as the Ustka harbour channel the driving force for the secondary bacterial production is also the river inflow of organic matter, which influences the zone where the channel enters the sea as well, but to the least extent.

The results of the present study showed significant seasonal dynamics in the total number of bacteria inhabiting the port channel in Ustka showed. The maximum total number of neustonic and planktonic bacteria was recorded in summer and the minimum in autumn and winter. These findings are consistent with the results of the studies from the estuarine and marine waters (Varela et al. 2003; Mudryk and Skórczewski 2007; Dreshchinskii and Engel 2017; Figueroa et al. 2021) and freshwater basins (Kalwasińska and Donderski 2005; Kostrzewska-Szlakowska and Kiersztyn 2017; Krevš et al. 2019). According to these researchers, the main stimulator of bacterial growth in summer is the intensive development of phytoplankton, often in bloom form. These organisms release organic matter to water in the form of assimilates, which are an optimal food base generating intensive multiplication and thus an increase in the number of bacteria at this time of year (Kalwasińska and Donderski 2005; Antonowicz et al. 2015). Summer maxima of the total number of bacteria in the examined channel may also be the effect of relatively high temperatures at this time of year (Mudryk and Skórczewski 2007; Zdanowicz and Mudryk 2017; Krevš et al. 2019). According to Benner et al. (1995) and Cottrell and Kirchman (2000) temperature is a major abiotic factor that significantly influences the seasonal variation in bacterial abundance in aquatic ecosystems.

In the port channel in Ustka, the seasonal differences in the level of bacterial production were noted. In eutrophicated ecosystems, the growth rate of bacteria and their production are mainly regulated by temperature, because the availability of food substrates produced mainly by phytoplankton in these water bodies is still very high (Ameryk et al. 2005). According to Gertman et al. (2013) and Yucel (2017) temperature due to its influence on all chemical and biochemical processes in cells is a key parameter regulating bacterial growth and their productivity at a seasonal scale. Spring and summer maxima in the level of bacterial production recorded in the studied water basin may be the effect of relatively high temperature at this time of year. These data correspond to the results of the studies by Shiah et al. (2000), Mudryk and Skórczewski (2007), Zdanowicz and Mudryk (2017), and Yucel (2017) in marine and inland reservoirs.

## Conclusion

Results presented in this study may add information in the explanation of the role of bacteriocenosis inhabiting the surface and subsurface layers of water of a polluted estuarine harbour channel in the degradation of organic matter and its flow through the microbial loop, which is a basic condition for maintaining homeostasis in any water body. However, although determination of bacterial numbers and their productivity in an estuary are methodically difficult processes, future studies need to be carried out as long-term investigations may provide reliable information on the role of bacteria as a key link in estuarine and marine biocenosis.

## Data Availability

All data generated or analysed during this study are included in this published article.
